# A hierarchical and modular approach to the discovery of robust associations in genome-wide association studies from pooled DNA samples

**DOI:** 10.1186/1471-2156-9-6

**Published:** 2008-01-14

**Authors:** Paola Sebastiani, Zhenming Zhao, Maria M Abad-Grau, Alberto Riva, Stephen W Hartley, Amanda E Sedgewick, Alessandro Doria, Monty Montano, Efthymia Melista, Dellara Terry, Thomas T Perls, Martin H Steinberg, Clinton T Baldwin

**Affiliations:** 1 Department of Biostatistics, Boston University School of Public Health, Boston 02118 MA, USA; 2 Department of Software Engineering, University of Granada, Granada 18071, Spain; 3 Department of Molecular Genetics, University of Florida at Gainesville, Gainesville 32611 FL, USA; 4 Bioinformatics Program, Boston University School of Engineering, Boston 02116 MA, USA; 5 Joslin Diabetes Center, Harvard Medical School, Boston 02215 MA, USA; 6 Department of Medicine, Boston University School of Medicine, Boston 02118 MA, USA; 7 Geriatric Section, Boston Medical Center, Boston 02118 MA, USA

## Abstract

**Background:**

One of the challenges of the analysis of pooling-based genome wide association studies is to identify authentic associations among potentially thousands of false positive associations.

**Results:**

We present a hierarchical and modular approach to the analysis of genome wide genotype data that incorporates quality control, linkage disequilibrium, physical distance and gene ontology to identify authentic associations among those found by statistical association tests. The method is developed for the allelic association analysis of pooled DNA samples, but it can be easily generalized to the analysis of individually genotyped samples. We evaluate the approach using data sets from diverse genome wide association studies including fetal hemoglobin levels in sickle cell anemia and a sample of centenarians and show that the approach is highly reproducible and allows for discovery at different levels of synthesis.

**Conclusion:**

Results from the integration of Bayesian tests and other machine learning techniques with linkage disequilibrium data suggest that we do not need to use too stringent thresholds to reduce the number of false positive associations. This method yields increased power even with relatively small samples. In fact, our evaluation shows that the method can reach almost 70% sensitivity with samples of only 100 subjects.

## Background

The availability of genotyping assays for hundreds of thousands of single nucleotide polymorphisms (SNP)s is making genome wide association (GWA) studies more accessible to a broad range of genotype-phenotype investigations. The promise of this technology is that it will accelerate gene discovery for polygenic diseases and complex phenotypes of Mendelian disorders because data for all genes can be obtained simultaneously [1,2]. At the same time, the large number of significance tests performed is expected to result in a large number of false positive association signals. In fact, the number of signals observed by chance may well be greater than those that are authentic [3]. Thus, the development of analytic methods and strategies to distinguish authentic signals from those due to chance will contribute significantly to disease-gene association studies.

Here we describe a modular procedure to analyze data from pooling-based GWA studies that use the Illumina SNP microarray technology [4]. Rather than genotyping individual samples, the pooling-based technology types a carefully constructed pool of DNA samples that can be used to infer allele frequencies and is an affordable alternative to GWA studies that are still a financial burden for many investigators. Several studies have shown the usefulness of pooling-based GWA studies to discover SNPs associated with disease [5-9] using well calibrated methods [7,10-12], and a variety of methods to estimate allele frequencies from pooled-based studies that use the Affymetrix microarray technology have been proposed [13,14]. Our objective is twofolds: (i) we wish to assess reproducibility and accuracy of the algorithm proposed by Illumina to detect chromosomal aberrations when used to estimate allele frequencies from pooled DNA samples [15]; and (ii) we propose a modular approach to the analysis of pooling-based GWA studies that limits the loss of power due to both the use of pools of DNA samples and the issue of multiple comparisons.

Several studies apply stringent thresholds on the significance level that is required to determine significant SNP-phenotype associations [16-18]. Contrary to this approach, our method integrates Bayesian tests for general associations [19] with decision rules based on the structure of linkage disequilibrium (LD) discovered through the International HapMap project [20], and other machine learning techniques to reduce the number of false positive associations. We also describe a hierarchical procedure to summarize the findings in terms of genes that can be further synthesized into gene sets using Gene Ontology annotations [21], pathways [22,23], or chromosomal bands. We evaluate this method using data from the sixty unrelated CEPH parents used for the International HapMap project [20] and two independent datasets. The first is a study of fetal hemoglobin (HbF) levels in African American subjects with sickle cell anemia and the objective is to discover genetic modulators of HbF. The second dataset is a study of exceptional longevity in a cohort of centenarians. In both datasets, using our novel analytic approach, we identified association signals in genes previously known to affect these phenotypes. The method is implemented in the R package and can be integrated with other R packages for genetic analysis, or GWA studies [24,25]. We develop the method for the analysis of pooled DNA samples [26,27], but the approach can be easily extended to the analysis of samples that are individually genotyped.

## Results

We ran three sets of experiments to assess the reproducibility and accuracy of the estimates of the allele frequencies derived from pooled DNA samples, as well as the sensitivity and specificity of our modular procedure.

### Experiment 1: accuracy and reproducibility

**Figure 1:**
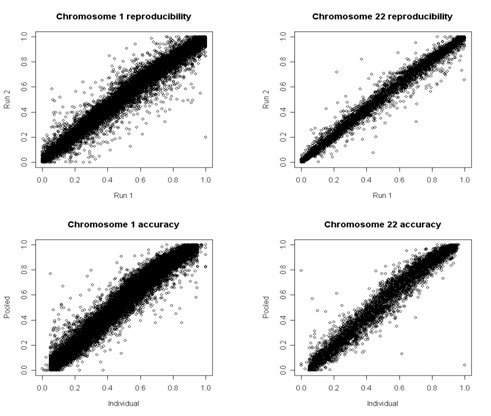
The reproducibility of the allele frequency estimates is shown by the scatter plot of repeated estimates of allele frequency inferred from pooled DNA samples (left). The labels "run 1" and "run 2" in the x- and y-axis specify each replication. The accuracy of the allele frequency estimates is shown by the scatter plot of the estimates of allele frequency inferred from pooled DNA samples (y-axis in the right plots) and those computed from individually genotyped samples (x-axis). The analysis of the other chromosomes shows similar results.

We obtained DNA samples from the 60 unrelated parents used in the HapMap CEU panel and created 2 duplicated pools of 30, 45 and 60 samples each (Table 1 provides a summary). The pooled DNA samples were analyzed in duplicates with the Illumina Sentrix HumanHap300 Genotyping BeadChip (v.1) and b-allele frequencies were estimated using the Illumina LOH and Copy Number analysis tool. The reproducibility was assessed by the agreement between allele frequency estimates in the two replicate samples for each pool (Table 1). Shown in Figure 1 is the scatter plot of two independent replicates of allele frequency estimates for the 22842 SNPs tagging chromosome 1 (top), and the 5452 SNPs tagging chromosome 22 (bottom) obtained with pools of 60 samples. The plots show a high degree of agreement that is confirmed for different sample sizes as shown by the results summarized in Table 1. Plots for other chromosomes are in the supplementary material [28].

**Table 1:** Summary of the results of Experiment 1.

Number of pools	Sample size	Average difference	Standard deviation	Correlation	Average error	Standard deviation	Correlation
2	30	0.0043	0.0303	0.9940	0.0304	0.0565	0.9860
2	45	0.0011	0.0295	0.9956	0.0331	0.0573	0.9890
2	60	0.0164	0.0498	0.9873	0.0451	0.0668	0.9890

Column 1: pool description; Column 2: number of samples per pool; Column 3: average difference between estimates of allele frequencies in repeated pools; Column 4: standard deviation of differences; Column 5: correlation between repeated allele frequency estimation. Column 6: average difference between estimates of allele frequencies from pooled DNA samples and individually genotyped samples. Column 7: standard deviation of the differences; Column 8: Correlation between estimates of allele frequencies from pooled DNA samples and individually genotyped samples.

We assessed the accuracy of the allele frequency estimates from pooled DNA samples by comparing the average estimates over the replicated pools with the allele frequencies computed using individually genotyped DNA samples that are available from the web site of the HapMap project [29]. A scatter plot of part of the results is displayed in Figure 1 for pools of 60 samples. The error analysis summarized in Table 1 suggests that, on average, the allele frequency based on the analysis of replicated pooled DNA samples differ from those based on individually genotyped data by approximately ± 0.04 but the error can be as large as ~0.12 = 0.04+2×0.06/√2 thus making differences in allele frequencies smaller than 0.24 difficult to detect because of technical errors. However, our analysis shows that less than 5% of the estimates based on pools of DNA samples differ from those based on individually genotyped samples by more than 0.12, and less than 10% differ by more than 0.08. This suggests reducing the minimum detectable allele frequency difference to 0.15 with a 10% chance of error. Furthermore, we have observed that amplifying DNA does not appear to affect either the reproducibility or the accuracy of the analysis.

To infer the effective sample size to be used in the analysis, we also looked at the distribution of the ratio between the two types of allele frequency estimates: say p(Si) = ni/n and q(Si) where ni is the frequency of the minor allele of the SNP Si computed from the samples that were typed individually, n is the overall sample size, and q(Si) is the frequency of the same minor allele computed from the analysis of the pooled DNA samples, in the different sets. The analysis demonstrated that log(q(Si)/p(Si)) has approximately a normal distribution with 0 mean and standard deviation 0.35. From this data, we deduced that about 95% of allele frequency estimates derived from the pooled DNA samples can be assumed to be within the interval p(Si) exp(± 1.95 × 0.35) from which we derive the empirical relation between p(Si) and q(Si): 0.51 p(Si) < q(Si) < 1.98 p(Si) with a range of uncertainty of 1.47 ni/n. The inequality suggests that when we infer allele frequency from pooled DNA samples, we have a loss of precision approximately equivalent to using 2/3 (= 1/1.47) of the original DNA sample size. We call this the "effective sample size" used in the calculation of the Bayesian test of association.

### Experiment 2: specificity

**Figure 2:**
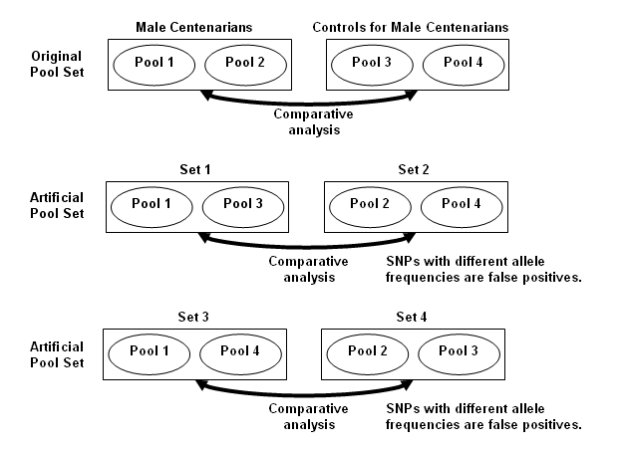
Example of the artificial pool sets that we created to assess the specificity of the procedure. As an example, the top four pools were generated to compare the genome of centenarians (pools 1 and 2) with that of younger controls (pools 3 and 4). The two artificial pool sets are obtained by mixing pools of centenarians DNA with those of controls.

**Figure 3:**
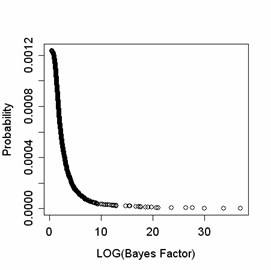
Distribution of the log_10 _Bayes factor in 1,114 false positive associations generated in approximately 10^6 ^association tests with an estimated false positive rate 5 10^-6^. The analysis shows that the chance to observe a very large Bayes factor has an exponential decay, and the probability of observing a Bayes factor greater than 10 by chance is 6 10^-4^, the probability of observing a Bayes factor greater than 100 is 3 10^-4^, greater than 1000 is 2 10^-4^.

To estimate the false positive rate (FPR) we used real data from pools of DNA samples to create artificial sets of pools. The original pools are described in Table 2 and were generated in duplicates to discover genetic variants associated with exceptional longevity [30], and fetal hemoglobin expression in subjects with sickle cell anemia [31]. The Illumina Sentrix HumanHap300 Genotyping BeadChip was used for all the experiments. We created the artificial sets of pools by mixing replicates of different pool sets. For example, we generated a set of two pools by taking one replicate of the pooled DNA samples from the female centenarians and one replicate of the pooled DNA samples from the younger female controls, and we constructed a second set of two pools by taking the remaining replicates from the two sets (See Figure 2 for an example). Because the two artificial sets of pools are homogeneous relative to the phenotype, the differences in allele frequencies between the two sets can be attributed to chance, and the SNPs with significant differences in allele distribution are false positives. We repeated this analysis by mixing different types of pools of DNA samples and using a BF>3, together with the LD and regional filters, we observed a false positive rate ranging between 0.001 and 4×10-4 with a mean of 0.001, and an average of 300 SNPs selected by chance. Note that this number is substantially smaller than the number of false positive associations that we would expect by chance using a BF>3. This threshold is equivalent to accepting an association when the posterior probability of the association is greater than 0.75, so that we expect 1 in 4 associations to be false. Also the specificity of the selected and significant genes was very high: The number of genes that by chance were selected in two unrelated analyses was 9 and this number was further reduced to 7 when we limited attention to significant genes. These numbers should provide a reference when we examine the reproducibility of findings in different studies, because we expect that, by chance alone, we would have an agreement in about 0.1% of findings. We note that long genes that are tagged by a larger number of SNPs are more likely to be selected by chance in different studies. Figure 3 displays the log10 BF in the 1,114 false positive associations generated in approximately the 106 association tests. The plot shows an exponential decay of the BF so that the chance to observe a very large BF has an exponential decay, and the probability of observing a BF greater than 10 by chance is 6 × 10-4, whereas the probability of observing a BF greater than 100 is 3 × 10-4, and greater than 1000 is 2 × 10-4. This analysis however shows that trying to reduce the false positive rate by imposing a stringent threshold on the BF would likely reduce the power of relatively small association studies and require unrealistically large sample sizes.

**Table 2:** Summary of the pools of DNA samples that were used for the validation of the analytical method. Each pool was done in duplicates.

Phenotype	Sample size
Exceptional longevity	130 male centenarians
	130 male controls
	130 female centenarians
	100 female controls
Fetal hemoglobin expression	55 sickle cell anemia subjects with fetal hemoglobin below 3% of the total hemoglobin
	54 sickle cell anemia subjects with fetal hemoglobin above 6.5% of the total hemoglobin

We also run experiments to assess the effect of LD and regional filters on the specificity. Using the same simulated sets, we run the analysis by using only a BF>3 to select the significant SNPs, and also examined the effect of adding either the LD filter or the regional filter or both on the false positive rate. Our results suggest that the LD filter reduces the false positive rate by 43%, while the regional filter alone increases the false positive rate by approximately 25%, and both filters decrease the false positive rate by 20%. These results are consistent with the intuition that the regional filter increases the power by finding clusters of SNPs that individually have small effects and would be disregarded by a one-SNP-at-a-time analysis. However, the effect is to slightly increase the false positive rate. This conjecture is confirmed in the next experiments that we conducted to assess the sensitivity.

### Experiment 3: sensitivity

In related work we are analyzing pools of DNA samples as a screening tool to discover genetic variants associated with exceptional longevity [30], and fetal hemoglobin (HbF) expression in subjects with sickle cell anemia [31]. As an indication of the sensitivity of technology and analytic method, we searched for SNPs in the Illumina Sentrix HumanHap300 Genotyping BeadChip that have been reported associated with either trait in independent studies, and verified whether an association was found based on the pooled DNA samples.

#### HbF experiment

We created two pools using DNA samples from 55 patients in the top and 54 patients in the bottom quartile of HbF concentrations. These patients were part of a clinical trial described in [32]. The pools were run in duplicates, and the data analyzed using the method proposed here. We searched the literature and found 36 SNPs with rs numbers that were reported associated with different levels of HbF [31,33-35]. Thirteen of these SNPs are in the Illumina array, and only 3 of these were found associated in our analysis with a BF greater than 1, and 2 with a BF greater than 3. The moderate effect of the other 10 SNPs (odds ratios between 0.55 and 1.76) is consistent with the weak associations reported by other investigators and would not be detectable with our sample size of about 60 subjects per group. In fact, a sample size of 60 subjects would give at most 30% power to detect an odds ratio of 1.75 when the MAF in one group is 0.5. We also found 23 SNPs in the Illumina array that are within 150 kb of the other 23 reported SNPs, and are associated with HbF levels with a BF greater than 1, and 13 of these had a BF greater than 3. Fifteen of these SNPs were typed as part of the HapMap project and ten of these are in strong LD (Bayes D' > 0.8)[36]. Thus the analysis based on pooled DNA samples discovered association of 26 SNPs, and for 13 of them the association was strong. This analysis suggests a sensitivity of 72% and if we limit attention to associations supported by a BF of at least 3 the sensitivity is 36%. The details of the associations are in Table 3.

**Table 3:** List of SNPs that are known to be associated with different levels of HbF and results of the analysis based on pooled DNA samples.

Number	SNP	Band	Gene	Validated	Distance	D'	BF	P_H	P_L	OR
**1**	**rs1143637**	**2q13**	***IL1B***	**rs12469600**	**16976**	**NA**	**1.39**	**0.13**	**0.04**	**3.08**
**2**	**rs31481**	**5q23.3**	***IL3***	**rs40401**	**724**	**1.00**	**2.32**	**0.48**	**0.65**	**0.48**
**3**	**rs271158**	**6q23.2**		**rs271156**	**641**	**1.00**	**22.86**	**0.53**	**0.77**	**0.35**
**4**	**rs454877**	**6q23.2**	***EYA4***	**rs211433**	**475**	**NA**	**2.43**	**0.49**	**0.32**	**2.09**
**5**	**rs212770**	**6q23.2**	***EYA4***	**rs11154727**	**4274**	**NA**	**1.25**	**0.12**	**0.04**	**3.03**
6	rs2295199	6q23.2		rs2295199	0		0.51	0.83	0.89	0.58
**7**	**rs210948**	**6q23.2**	***MYB***	**rs210798**	**17242**	**NA**	**2.17**	**0.78**	**0.90**	**0.38**
**8**	**rs509342**	**6q23.2**	***PDE7B***	**rs560713**	**23645**	**0.95**	**1.47**	**1.00**	**0.95**	**17.80**
**9**	**rs2076192**	**6q23.3**	***MAP7***	**rs2076193**	**44438**	**0.97**	**1.30**	**0.52**	**0.37**	**1.88**
**10**	**rs997139**	**6q23.3**	***MAP7***	**rs3799419**	**2100**	**1.00**	**1.35**	**0.06**	**0.14**	**0.36**
**11**	**rs3778314**	**6q23.3**	***MAP7***	**rs2181096**	**25746**	**0.89**	**57.04**	**0.61**	**0.85**	**0.28**
**12**	**rs2237262**	**6q23.3**	***MAP3K5***	**rs3799472**	**18405**	**0.98**	**1.03**	**0.62**	**0.76**	**0.53**
**13**	**rs2012700**	**6q23.3**	***PEX7***	**rs2012700**	**0**	**0**	**1.50**	**0.34**	**0.50**	**0.52**
14	rs717088	6q23.3	*PEX7*	rs717088	0		0.47	0.37	0.47	0.65
**15**	**rs3799476**	**6q23.3**	***PEX7***	**rs3799479**	**44021**	**NA**	**1.98**	**0.12**	**0.25**	**0.42**
16	rs1342645	6q23.3	*PEX7*	rs1342645	0		0.67	0.77	0.86	0.55
**17**	**rs1342641**	**6q23.3**		**rs1342642**	**22583**	**0.06**	**6.12**	**0.96**	**0.84**	**4.98**
**18**	**rs1322393**	**6q23.3**	***IL20RA***	**rs1322394**	**661**	**1.00**	**3.00**	**0.23**	**0.40**	**0.44**
**19**	**rs44450**	**6q23.3**		**rs276568**	**3229**	**1.00**	**47.32**	**0.37**	**0.64**	**0.34**
20	rs1349115	8q12.1	*TOX*	rs1349115	0		0.69	0.29	0.41	0.59
21	rs10504269	8q12.1	*TOX*	rs10504269	0		0.61	0.72	0.82	0.58
22	rs6997859	8q12.1	*TOX*	rs6997859	0		0.37	0.23	0.17	1.45
23	rs12155519	8q12.1	*TOX*	rs12155519	0		0.94	0.58	0.44	1.77
**24**	**rs1947178**	**8q12.1**	***TOX***	**rs1947178**	**0**		**21.86**	**0.10**	**0.29**	**0.28**
25	rs746867	8q12.1	*TOX*	rs746867	0		0.29	0.29	0.35	0.76
26	rs389349	8q12.1	*TOX*	rs389349	0		0.42	0.95	0.93	1.43
**27**	**rs851800**	**8q12.1**	***TOX***	**rs396720**	**23622**	**0.15**	**9.20**	**0.28**	**0.11**	**3.24**
**28**	**rs380620**	**8q12.1**	***TOX***	**rs2561145**	**300069**	**0.58**	**10.67**	**0.08**	**0.24**	**0.26**
**29**	**rs2043190**	**9q34.11**	***ASS***	**rs540140**	**609**	**NA**	**19.56**	**0.99**	**0.88**	**24.90**
**30**	**rs7482144**	**11p15.4**	***HBG2***	**rs3813727**	**20257**	**NA**	**2.52**	**0.17**	**0.32**	**0.43**
**31**	**rs723623**	**15q13.3**	***C15orf16***	**rs6493688**	**8825**	**0.51**	**1.31**	**0.93**	**0.84**	**2.66**
32	rs1867380	15q22.31	*AQP9*	rs1867380	0		0.28	0.82	0.84	0.85
**33**	**rs4489951**	**15q22.31**	***MAP2K1***	**rs4489951**	**0**		**17.66**	**0.36**	**0.60**	**0.38**
**34**	**rs1440372**	**15q22.31**	***SMAD6***	**rs2469141**	**65753**	**0.03**	**2181.00**	**0.68**	**0.33**	**4.24**
**35**	**rs8038623**	**15q22.31**	***SMAD3***	**rs6494633**	**16831**	**NA**	**87.23**	**0.88**	**0.65**	**4.09**
**36**	**rs2227319**	**17q21.1**	***CSF3***	**rs2071369**	**1460**	**1.00**	**3.42**	**0.90**	**0.77**	**2.88**

Column 1: row number; Column 2: SNP ID; Column 3: Cytogenic band; Column 4: Genes tagged by the SNP; Column 5: SNP in the Illumina array that was used to compare the association. If the SNP to be validated was not in the array, we searched for the closest SNP within 100 kb from that to be validated with a positive Bayes Factor; Column 6: distance between the two SNPs; Column 7: Bayes D' between the two SNPs, an NA means that the SNPs originally reported as associated with HbF is not in the HapMap data. Column 8: Bayes Factor; Column 9–10: estimates of allele frequencies in the pools of DNA from patients with high HbF and low HbF; Column 11: Odds ratio. The SNPs 6, 13, 14, 16, 20–26, 32 and 33 are in the array and SNPs 13, 24 and 33 were found associated with different levels of HbF. Highlighted in bold are the associations confirmed by our analysis.

#### Longevity experiment

We created pools of DNA samples from unrelated centenarians and younger controls. Because there is evidence of gender effect [37] --- 85% of centenarians are female--- we created distinct pools for males and females as summarized in Table 2. We searched the literature and found 36 SNPs with rs numbers reported as associated with longevity [38-40]. Seven of these 36 SNPs are in the Illumina array, and five of these seven were found associated in our analysis. For 21 of the remaining 29, we found SNPs within 100 kb that were associated with longevity in either the males and female comparisons, or both. These SNPs are reported in Table 4. The analysis suggests approximately 67% sensitivity, and this is consistent with the sensitivity estimated with the HbF experiment. We also noted that the regional filter helped identify some of the associations that would be lost with a tight threshold on the BF. As an example, the SNP rs2227956 on HSPA1A was found associated with longevity in males only when the regional filter is used, and the two SNPs in WRN -a well known longevity gene in mice [41] – were selected by the regional filter. A similar form of sensitivity analysis is to see whether the GSEA analysis can lead to discover sets of functionally similar genes that are known to be associated with longevity. GSEA analysis of the centenarian cohort revealed several enriched GO biological categories (Table 4 and manuscript in preparation). Among the significantly enriched categories were genes associated with immune response (e.g., CSF3) and DNA repair (e.g., XRCC4), see Table 4. Intriguingly, CSF3 (also known as GCSF) is reported to influence migration of stem cells between the bone marrow and blood [42,43] and appears to promote regeneration of myocardial tissue [44-46], which has clear relevance to longevity. The gene XRCC4 has a well established role in DNA repair [47], and unrepaired DNA has been reported to accelerate ageing, possibly through dysregulating the IGF/growth axis [48]. Therefore, a comprehensive analysis of these and other genes present with the enriched gene sets that we detected will be essential to fully appreciate pathways engaged that contribute to the longevity phenotype.

**Table 4:** List of SNPs that were found associated with exceptional longevity in different studies, and results based on the analysis of pooled DNA samples.

				Males							Females						
Index	SNP	Band	Gene	Validated	Distance	D'	BF	P_L	P_C	OR	Validated	Distance	D'	BF	P_L	P_C	OR

**1**	**rs1870377**	**4q12**	***KDR***	**rs2305945**	**1128**	**1.00**	**1.5**	**0.84**	**0.75**	**1.71**	**rs2305945**	**1128**	**1.00**	**1.1**	**0.77**	**0.68**	**1.62**
**2**	**rs2866164**	**4q23**	*MTP*	**rs7693203**	**9187**	**NA**	**3.4**	**0.82**	**0.72**	**1.83**	**rs1057613**	**14042**	**NA**	**8.6**	**0.64**	**0.49**	**1.90**
**3**	**rs750032**	**4q24**	*PPP3CA*	**rs2850971**	**3819**	**NA**	**41.4**	**0.87**	**0.74**	**2.39**	**rs2850971**	**3819**	**NA**	**10.8**	**0.82**	**0.69**	**2.12**
**4**	**rs951085**	**4q24**		rs9999238	29363	0.98	0.2	0.81	0.83	0.85	**rs9999238**	**29363**	**0.98**	**10.9**	**0.81**	**0.67**	**2.09**
**5**	**rs28360135**	**5q14.2**	*XRCC4*	**rs1382367**	**40462**	**NA**	**295053.4**	**0.32**	**0.59**	**0.33**	**rs1382367**	**40462**	**NA**	**2.6**	**0.50**	**0.63**	**0.58**
**6**	**rs1799945**	**6p22.2**	*HFE*	**rs1572982**	**3188**	**0.99**	**28228.6**	**0.67**	**0.42**	**2.78**	**rs1572982**	**3188**	**0.99**	**11.2**	**0.44**	**0.61**	**0.51**
**7**	**rs9380254**	**6p21.33**	*MICA*	**rs1131896**	**780**	**1.00**	**5744.1**	**0.67**	**0.87**	**0.32**	rs1131896	780	1.00	0.8	0.83	0.84	0.96
**8**	**rs2227956**	**6p21.33**	*HSPA1L*	**rs2227956**	**0**		**12.6**	**0.10**	**0.20**	**0.42**	rs2227956	0		0.3	0.18	0.14	1.31
**9**	**rs1800797**	7p15.3	*IL6*	rs2056576	5019	0.64	0.2	0.70	0.72	0.91	**rs2056576**	**5019**	**0.99**	**1.7**	**0.75**	**0.64**	**1.70**
**10**	**rs662**	7q21.3	*PON1*	rs662	0		0.2	0.33	0.29	1.21	**rs662**	**0**		**2.8**	**0.32**	**0.21**	**1.77**
**11**	**rs1799983**	**7q36.1**	*NOS3*	**rs2373929**	**18701**	**0.16**	**6.1**	**0.50**	**0.64**	**0.57**	rs2373929	18701	0.07	0.6	0.63	0.54	1.45
**12**	**rs2251621**	**8p12**	*PURG*	**rs13269094**	**8189**	**0.66**	**1936.2**	**0.07**	**0.22**	**0.26**	**rs1362911**	**23018**	**0.47**	**>10^6**	**0.69**	**0.96**	**0.10**
**13**	**rs2725362**	**8p12**	*WRN*	**rs3024239**	**158**	**NA**	**4942.7**	**0.34**	**0.57**	**0.39**	**rs2725362**	**0**		**27.2**	**0.48**	**0.66**	**0.48**
**14**	**rs1346044**	**8p12**	*WRN*	**rs1346044**	**0**		**257.9**	**0.36**	**0.19**	**2.44**	rs1346044	0		0.4	0.28	0.21	1.46
**15**	**rs5744256**	**11q23.1**	*IL18*	**rs243908**	**16909**	**0.61**	**>10^6**	**0.63**	**0.29**	**4.02**	rs243908	16909	0.96	0.2	0.28	0.28	1.01
**16**	**rs675**	**11q23.2**	*APOA4*	**rs581015**	**5559**	**0.28**	**1.3**	**0.61**	**0.51**	**1.55**	**rs10502189**	**371**	**1.00**	**4.8**	**0.05**	**0.00**	**56.22**
**17**	**rs1467558**	**11p33**	*CD44*	**rs1467558**	**0**		**2.0**	**0.91**	**0.83**	**1.97**	rs1467558	0		0.2	0.84	0.86	0.87
**18**	**rs9536314**	**13**	*KL*	**rs9536314**	**0**		**5.1**	**0.24**	**0..36**	**0.55**	**rs9536314**	**0**		**99.0**	**0.36**	**0.18**	**2.56**
**19**	**rs861539**	**14**	***XRCC3***	rs861539	0		0.15	0.75	0.74	1.05	**rs861539**	**0**		**68.7**	**0.74**	**0.55**	**2.27**
**20**	**rs8052394**	**16q12.2**	***MT1A***	**rs7189840**	9798	NA	0.14	0.59	0.57	1.09	**rs7189840**	**9798**	**NA**	**54.1**	**0.56**	**0.37**	**2.18**
**21**	**rs1800776**	**16q13**	***CETP***	**rs3764261**	**1910**	**NA**	**1.1**	**0.59**	**0.69**	**0.65**	rs3764261	1910	NA	0.15	0.66	0.67	0.97
22	rs5882	16q13	*CETP*	rs5882	0		0.19	0.39	0.35	1.19	rs5882	0		0.17	0.37	0.40	0.88
**23**	**rs704**	**17q11.2**	***VTN***	**rs2027993**	**12085**	**0.99**	**17.3**	**0.45**	**0.61**	**0.53**	**rs2027993**	**12085**	**0.99**	**1.1**	**0.58**	**0.47**	**1.57**
**24**	**rs4344**	**17q23.3**	***ACE***	**rs4343**	**693**	**1.00**	**1.4**	**0.69**	**0.78**	**0.61**	**rs4343**	**693**	**1.00**	**6.4**	**0.66**	**0.51**	**1.87**
**25**	**rs2252673**	**19p13.2**	***INSR***	**rs2059807**	**15691**	**0.23**	**1.5**	**0.63**	**0.73**	**0.63**	rs2059807	15691	0.31	0.2	0.75	0.72	1.16
**26**	**rs1799782**	**19**	***XRCC1***	**rs9394611128**	**11001**	**NA**	**1.4**	**0.05**	**0.10**	**0.47**	**rs939461**	**11001**	**NA**	**12.6**	**0.09**	**0.02**	**5.88**

Column 1: row number; Column 2: SNP ID; Column 3: Cytogenic band; Column 4: Genes tagged by the SNP; Column 5: SNP in the Illumina array that was used to compare the association as described in the caption of Table 3; Column 6: distance between the two SNPs; Column 7: Bayes D' between the two SNPs; Column 8: Bayes Factor; Column 9–10: estimates of allele frequencies in the pools of DNA from male centenarians and younger controls; Column 11: Odds ratio. Columns 12–18: as columns 5–11 but for the pools comparing female centenarians to younger controls. The SNPs 8, 10, 14, 16–19, 22 are in the array and SNPs 8, 14, 17–19 were found associated with longevity in females and/or males. Highlighted in bold are the associations confirmed by our analysis.

## Discussion and Conclusion

We have developed a hierarchical and modular approach to the analysis of genome wide genotype data based on pooled DNA samples. The method incorporates quality control data, information about linkage disequilibrium, Bayesian association tests, physical distance and gene ontology to identify associations warranting further investigation. Our evaluation using real data has shown the accuracy, reproducibility, sensitivity and specificity of the method.

Compared to other approaches, the integration of Bayesian tests with information about linkage disequilibrium and other machine learning techniques implies that we do not need to use too stringent thresholds to reduce the number of false positive associations. The implication of this fact is an increased power even with relatively small samples. In fact, our estimate of the sensitivity shows that the method can reach almost 70% sensitivity with samples of only 100 subjects.

Although we developed the approach to analyze pooled DNA samples, the method can also be used for the analysis of individually genotyped samples.

## Methods

### Genotyping

For the HbF study, DNA was obtained from the 60 subjects with HbF levels below the first quartile of the distribution, and the 60 subjects with HbF levels above the third quartile who were enrolled in the Multicenter Study of Hydroxyurea (MSH) study in Sickle Cell Anemia [49]. DNA samples from 260 centenarians and a control group of 230 subjects were obtained from the New England Centenarian Study: a cross-sectional study of individuals aged 97 and older conducted at the Boston Medical Center. CEPH DNA samples of the sixty unrelated parents used for the International HapMap project [20] were obtained from the Coriell Institute, Camden, NY and used to compare the accuracy and reproducibility of the estimates of allele frequency in pooled DNA samples compared to individually genotyped samples. For DNA pool construction and to ensure that each individual contributed equally to the pool, we first measured DNA stock solutions using a fluorimetric method (RNAseP) against a standard curve constructed from known concentrations of human genomic DNA. We then diluted the stock solutions to 10 ng/ul and measured the concentrations of these working solutions by means of PicoGreen. In the case of samples for which the CV of the three measurements was greater than 10%, quantification was repeated in triplicate until the CV was smaller than 10. Measurements were highly reproducible, with a correlation coefficient of 0.97 between the third measurement and the average of the first two. Based on these concentrations, 50 ng of DNA were added to the pool for each individual. The pools of DNA were analyzed on the Sentrix HumanHap300 bead chip (Illumina) according to the manufacture's protocol. The data used in the HbF and longevity studies will be released with companion publications. We make available the data derived from pools of CEPH DNA samples from the supplementary web site [28]. The HbF and longevity studies were approved by the Institutional Review Boards of Boston University.

### Association test

**Figure 4:**
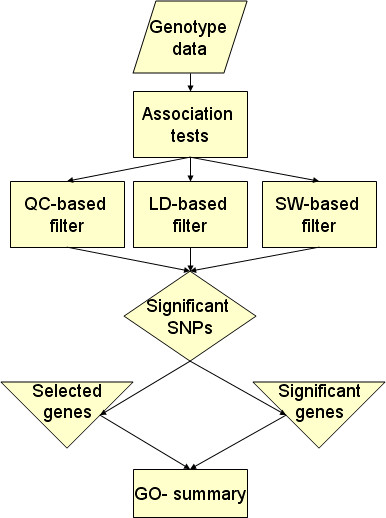
Schematic summary of the modular approach to the analysis of GWA data.

The overall analytic strategy is shown in Figure 4. The first module is a statistical procedure to test the allelic association between each individual SNP and the phenotype. The input data are the allele relative frequencies estimated with the "b-allele frequency" value provided by the Illumina Beadstudio genotype module. This value represents the relative proportion of each allele in the DNA sample and is used by the Illumina loss of heterozygosity (LOH) and Copy Number analysis tool [15] to detect chromosomal aberrations and copy numbers by comparing the normalized intensity of the test sample (the pooled DNA samples) to a reference sample. We use the estimate of the allele frequencies *θ*_*ij *_in test and control pools to reconstruct the expected allele frequencies as $n_{ij}=n_{i}^{*}\theta_{ij}$ where $n_{i}^{*}$ is the *effective sample size *in pool i, the index i = 1 for cases and i = 2 for controls, and the index j = A,B denotes the A or B allele. We then use a Bayesian test of association to compare the distributions of allele frequency in the two different pools. The test is described in [19,50] and assumes that prior probabilities are available for the model of allelic association and the model of no association – say *p*(*M*_*a*_), *p*(*M*_*i*_) – and then uses the data to update these prior probabilities *p*(*M*_*a*_), *p*(*M*_*i*_) into the posterior probabilities by using Bayes' theorem. The decision rule is then to select the model of association if its posterior probability is at least 3 times larger than the posterior probability of the model of no association (as suggested in reference [51]). Formally, the ratio of the posterior probabilities is

Bayestest :p(Ma|nij)p(Mi|nij)=p(nij|Ma)p(nij|Mi)×p(Ma)p(Mi)

and the ratio of the marginal likelihood functions *p*(*n*_*ij *_| *M*_*a*_)/*p*(*n*_*ij *_| *M*_*i*_) is known as the Bayes factor (BF). When the prior probabilities of the two models are equal, the BF is equivalent to the posterior odds. Assuming the conjugate Beta distribution for the allele frequencies, the BF can be calculated in closed form and the formula for this calculation is reported in the appendix. To take into account the issue of multiple testing, we can use prior information about the number of SNPs that we expected to be associated to make the selection stronger. For example, if we expect 1,500 SNPs associated with the phenotype, then the prior odds for the alternative hypothesis of association are 0.005/0.995 when we test 300,000 SNPs, and the decision rule becomes to accept that a SNP is associated with the phenotype if the posterior odds for the association are at least 3 × 0.995/0.005 = 597. Initial experiments described in the Evaluation section suggest that a robust choice for an effective sample size is 2/3 of the original pool, and this is consistent with a larger sample size needed with the analysis of pooled DNA samples [52]. We note here that one advantage of this modular procedure is that the Bayesian test can be replaced by a standard *χ*^2 ^test for allelic association.

### Filtering out false positives

Although we can take into account the issue of multiple comparisons by choosing appropriate prior odds for an association, the consequence of this approach is to reduce power and to require large sample sizes to detect associations with a small effect. This consequence can be problematic in studies where cases are relatively rare such as the study of exceptional longevity in which cases are subjects who lives 100 years and older. To fully exploit the power of small scale studies we developed a series of data filters that remove unreliable or suspicious associations (see Figure 4). The first filter is specific for allele association analysis using pooled DNA samples and accounts for the lack of precision of the technology. The other two filters take into account redundancy as well as reciprocal information of SNPs based on the LD structure of the human genome. So, rather than using "SNP pruning" as in PLINK to remove SNPs that are in LD [53], we leverage on dependencies determined by LD to improve the detection of false positive while reducing the false negative rate.

### Quality control (QC) filter

The function of this filter is based on an extensive evaluation of the accuracy and reproducibility of the allele frequency estimates that are computed with the Illumina software. Allele frequencies obtained from genotyping of pooled DNA samples were compared with those derived from genotyping of individual samples (detailed in the below Evaluation section). The results suggest that alleles with minor allele frequency MAF <0.15 as well as differences in allele frequency of less than 0.15 are not reliable. We therefore filter out all SNPs with these characteristics, as well as those SNPs for which repeated estimates of allele frequencies in replications of the same pool differ by more than 0.15.

### Linkage disequilibrium (LD) filter

SNPs in LD with each other would be expected to show similar patterns of association if the signal is authentic whereas a single SNP in a LD block showing association is more likely to represent a spurious association. Therefore, our procedure automatically checks this condition and disregards the associations for SNPs that are not supported by positive associations with other SNPs in the same LD block. To this end, we used genotype data collected within the HapMap project to compute pairwise measures of LD for all consecutive pairs of SNPs in the HumanHap300 platform. The estimation of LD was based on a novel Bayesian version of D' that we introduced in [36]. As the traditional D', our Bayesian estimator is defined in the interval [0;1] regardless of the allele frequency so that it is easier to interpret than other measures of correlation like r2 but it is much less biased toward disequilibrium. We use a Bayesian D' > 0.7 between pairs of consecutive SNPs as suggestive of strong LD and we filter out all the associations of the SNPs whose adjacent SNPs that are in strong LD are not associated with the phenotype. The value 0.7 was chosen based on experiments reported in [36] showing that the Bayesian D' rarely exceeds 0.7 under no LD. The Bayesian D' values for each pair of consecutive SNPs were built for Caucasians using the DNA samples from unrelated parents of thirty trios of the CEPH (Utah residents with ancestry from northern and western Europe, also known as CEU) and similarly for Africans, using Yoruba in Ibadan Nigeria. These data are available from the supplementary material web site [28].

### Regional association filter

**Figure 5:**
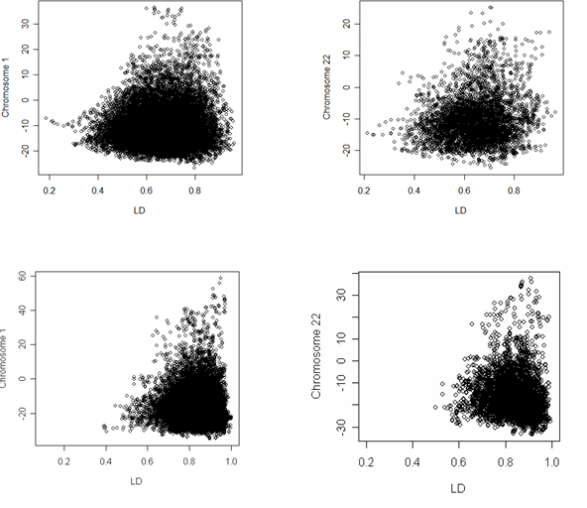
Relation between the pattern of LD (x-axis) and the global measure of association (y-axis) in the regional filter. The pattern of LD is measured by the average of the Bayes D' between consecutive SNPs in the region, and the global measure of association is the joint probability of association in the region. The two figures in the top half show the relation using data from the study of fetal hemoglobin in the sickle cell anemia subjects. The two figure in the bottom half show the relation using data from the longevity study. The different extent of LD reflect the fact that sickle cell anemia subjects are all African American while centenarians in the longevity study are all Caucasians The correlations in the four sets are 0.03, 0.18, 0.018, -0.10.

The rationale of this filter is that a region or gene showing authentic association would be expected to show a greater number of SNPs associated than would be expected by chance. In this filter, we analyze the data using a sliding window of 20 SNPs, and summarize the global measure of association within the window as the product of the posterior probabilities of associations of the 20 SNPs. Here, we assume that the association tests are independent, so that the product of the posterior probability of association of the individual SNPs becomes a measure of the global association of the region tagged by the 20 SNPs. Windows with a global measure of association exceeding 0.5 [or product of BFs >1] are then selected for further inspection. Although LD between SNPs in a window may introduce dependencies, the global measure of association does not seem to be affected by this approximation. Figure 5 shows some examples.

### Hierarchical summary

The list of SNPs that are selected by the association test and the filters are labeled as "significant SNPs". The list is annotated by the SNP physical position, the position relative to known genes, the allele frequencies estimated in different populations, and the cytogenetic band. This information is collected through SNPPer [54] that integrates information from the UCSC human genome browser and dbSNP [55]. As a further level of summary we use those SNPs that are linked to genes to create a set of selected genes and a set of significant genes. The first set consists of genes that are tagged by at least one significant SNP. The set of significant genes is a subset of the selected genes and consists of genes in which the global measure of association given by the product of the posterior probability of association of the gene tagging SNPs is greater than 0.5 [Or equivalently, the product of BFs exceeds 1].

### Ranking

We rank the significant genes by the global measure of association. To rank the selected genes we score them by two further measures that weigh the likelihood of selecting a gene by chance. In fact, there are genes that are tagged by a large number of SNPs: for example CSMD1 in chromosome 8 is tagged by 614 SNPs in the HumanHap300 array, and assuming a 5% false positive rate, we would expect about 30 SNPs to be selected from this gene by chance in any analysis. To take this issue into account, each selected gene is assigned 3 scores: the global measure of association, the ratio of selected SNPs relative to the number of tagging SNPs, and the probability of selecting this number by chance using the hyper-geometric distribution. Each score determines a ranking and then the sum of the ranks is defined as a final ranking of selected genes.

### Gene set enrichment analysis

To evaluate selected and/or significant genes for enrichment of biological categories associated with a variable phenotype, we implement a stand-alone version of the EASE statistical software [56]. This program computes a modified Fisher's exact probability score for observing the frequency of a biological category associated with a phenotype (e.g., dementia, sickle phenotype, infection), compared with the likelihood of identifying that category by chance given the total number of genes in the data set. An adjusted score is then reported representing the upper bound of the distribution of Jackknife Fisher exact probabilities for observing an enriched biological category. Enriched categories are then inspected for biological trends and overlapping or related categories, based on significance scores or categories with a p-value << 0.05. For more detail, see Hosack et al. [56].

## Authors' contributions

PS developed and implemented the analytic method, conceived the evaluation and drafted the manuscript. ZZ contributed to the implementation and evaluation. MAG, AR, SH, AS, developed the support material for the various filters and hierarchical summary. AD helped to design and conduct the experiments with the CEPH samples. MM contributed to the development of the hierarchical method and the interpretation of the analysis results. EM and CB carried out the pooling-based GW genotyping. DT, TTP, MS contributed to the development and evaluation of the method using the longevity study and the HbF study. All authors read and approved the final manuscript.

## Appendix

### Derivation of the Bayes Factor

This Bayesian association test assumes that the allele frequencies follow a binomial distribution with probabilities *θ*_*ij *_the index i = 1 for cases and i = 2 for controls, and the index j = A,B denotes the A or B allele [See Table 5 for an example]. Under the hypothesis of general association, the parameters *θ*_*ij *_describing the allele distributions in cases and controls follow different probability distributions, while the parameters *θ*_*ij *_follow the same probability distribution under the hypothesis of no association. Therefore, the likelihood function under the hypothesis of general association *M*_*a *_is

**Table 5:** parameters and allele frequencies from pooled DNA samples

	Allele A	Allele B	
Cases (one pool)	n_1A _= p(A|cases)*(2n_1_)	n_1B _= p(B|cases)*(2n_1_)	2n_1_
Controls (one pool)	n_2A _= p(A|controls)*(2n_2_)	n_2B _= p(B|controls)*(2n_2_)	2n_2_
	n_A_	n_B_

$$M_{a}:p(\theta_{ij}|n_{ij})\propto \theta_{1A}^{n_{1A}}{\left(1-\theta_{1A}\right)}^{n_{1B}}\theta_{2A}^{n_{2A}}{\left(1-\theta_{2A}\right)}^{n_{2B}}$$

While the likelihood function under the hypothesis of no association *M*_*i *_is:

$$M_{i}:p(\theta_{ij}|n_{ij})\propto \theta_{A}^{n_{A}}{\left(1-\theta_{A}\right)}^{n_{B}}$$

We assume independent Beta distributions to model the prior distributions of the parameters that are defined as

$$\begin{array}{l} \begin{array}{cc} M_{a}:p(\theta_{ij})\propto \theta_{1A}^{\alpha_{1A}-1}{\left(1-\theta_{1A}\right)}^{\beta_{1B}-1}\theta_{2A}^{\alpha_{2A}-1}{\left(1-\theta_{2A}\right)}^{\beta_{2B}-1} & \text{and} \end{array}\\ M_{i}:p(\theta_{ij})\propto \theta_{A}^{\alpha_{A}-1}{\left(1-\theta_{A}\right)}^{\beta_{B}-1} \end{array}$$

Where the hyper-parameters are chosen as *α*_1*A *_= *α*_2*A *_= *α*_*A*_/2 = *α*/4 and

*β*_1*B *_= *β*_2*B *_= *β*_*B*_/2 = *α*/4. The parameter *α *is the overall prior precision and can be set based on prior information. The likelihood function and the prior distribution of the parameters are used to compute the marginal likelihood as the expected likelihood function, where the expectation is taken over the parameter distribution. Formally

*p*(*M *| *n*_*ij*_) = ∫*p*(*θ*_*ij *_| *n*_*ij*_)*p*(*θ*_*ij*_)*dθ*_*ij*_

Compared to the maximum likelihood that returns the likelihood function evaluated in the estimate of the parameters, the marginal likelihood incorporates the uncertainty about the parameters by averaging the likelihood functions for different parameter values. This conceptual difference is fundamental to understand the different approach to model selection: in the classical framework, model selection is based on the maximized likelihood and its sampling distribution to take into account sampling variability, for fixed parameter values. In the Bayesian framework, model selection is based on the marginal likelihood which takes into account the parameter variability, for fixed sample values. Therefore, no significance testing is performed when using this approach to model selection. Our experience with the Bayesian procedure to model selection is that it is usually more robust to false associations. The calculation of the marginal likelihood can be done in closed form and it is easy to show that

$$p(M_{a}|n_{ij})=\frac{\Gamma (\alpha_{1A}+\beta_{1B})\Gamma (\alpha_{2A}+\beta_{2B})}{\Gamma (\alpha_{1A}+\beta_{1B}+n_{1})\Gamma (\alpha_{2A}+\beta_{2B}+n_{2})}\times \frac{\Gamma (\alpha_{1A}+n_{1A})\Gamma (\beta_{1B}+n_{1B})\Gamma (\alpha_{2A}+n_{2A})\Gamma (\beta_{2B}+n_{2B})}{\Gamma (\alpha_{1A})\Gamma (\alpha_{2A})\Gamma (\beta_{1B})\Gamma (\beta_{2B})}$$

$$p(M_{i}|n_{ij})=\frac{\Gamma (\alpha )}{\Gamma (\alpha +n)}\frac{\Gamma (\alpha_{1A}+\alpha_{2A}+n_{A})\Gamma (\beta_{1B}+\beta_{2B}+n_{B})}{\Gamma (\alpha_{1A}+\alpha_{2A})\Gamma (\beta_{1B}+\beta_{2B})}$$

Where G is the gamma function and the ratio produces the Bayes factor.
